# Content Validation through Expert Judgement of an Instrument on the Nutritional Knowledge, Beliefs, and Habits of Pregnant Women

**DOI:** 10.3390/nu12041136

**Published:** 2020-04-18

**Authors:** Elisabet Fernández-Gómez, Adelina Martín-Salvador, Trinidad Luque-Vara, María Angustias Sánchez-Ojeda, Silvia Navarro-Prado, Carmen Enrique-Mirón

**Affiliations:** 1 Department of Nursing, Faculty of Health Sciences, Melilla Campus, University of Granada, Calle Santander s/n, 52001 Melilla, Spain; elisabetfdez@ugr.es (E.F.-G.); ademartin@ugr.es (A.M.-S.); maso@ugr.es (M.A.S.-O.); silnado@ugr.es (S.N.-P.); 2 Department of Inorganic Chemistry, HUM-613 Research Group, Faculty of Health Sciences, Melilla Campus, University of Granada, Calle Santander s/n, 52001 Melilla, Spain; cenrique@ugr.es

**Keywords:** content validity, expert judgement, Fleiss’ *κ*, pregnant women, eating habits

## Abstract

The aim of this study was to conduct content validation through expert judgement of an instrument which explores the nutritional knowledge, beliefs, and habits during pregnancy. This is a psychometric study in which 14 experts participated in the evaluation of each of the questionnaire items, which were divided into two blocks according to the characteristics of sufficiency, clarity, coherence, and relevance. Fleiss’ κ statistic was used to measure strength of agreement. A pre-test with 102 participants was conducted to measure the degree of understandability of the instrument. The strength of agreement obtained for each of the dimensions was almost perfect. For each pair of experts, strength of agreement ranged between substantial and almost perfect. Sufficiency was the characteristic of the questionnaire that obtained the highest values in the two blocks, and was also the most statistically significant (*p < 0*.001). Coherence was the most statistically significant characteristic in the first block (*p* = 0.030). Clarity was the most statistically significant characteristic in the second block (*p* = 0.037). The wording of five of the twenty original items was corrected. The new version of the instrument attained a high degree of understandability. The results suggest that the instrument is valid and may therefore be applied.

## 1. Introduction

Studies involving women during pre-conception, pregnancy, and breastfeeding report inadequate food intake for their physiological state and highlight the real need to ensure proper maternal and fetal nutrition [1]. Inadequate maternal nutritional intake during pregnancy may lead to adverse outcomes in fetal development, such as negative metabolic effects on offspring [2]. Pregnant women’s nutritional knowledge may influence their food intake [3]. This is why it is considered of paramount importance to be able to delve into the nutritional knowledge and beliefs pregnant women have, as intended in the present study.

There is currently no standard guideline for validating health-related measures. However, several criteria developed in the fields of psychology and education sciences are used. There is a growing need for the use of health-related measuring instruments in clinical and research practice. The methodology for the adaptation of instruments is not very well known among health professionals, which may explain the existence of incomplete instruments or word-for-word translations of existing instruments in the field. A number of relevant and essential skills and guidelines, which should be acquired and implemented in a dynamic and continuous way, are therefore required for the validation of an instrument [4].

Reliable instruments are necessary, as are instruments that have been validated using construct validity, criterion validity, and content validity, which are the most widely used measures of validity [5]. Validity and reliability are two quality criteria that any measuring instrument must satisfy in order to be used by researchers in their studies. In order to validate health-related measuring instruments and ensure that they are reliable and valid, their psychometric properties must be subjected to a process of adaptation and validation. All of this is essential to determine the quality of the measurements produced by these instruments [4]. Content validity is defined as, “the degree to which elements of an assessment instrument are relevant to, and representative of, the targeted construct for a particular assessment purpose” [6] (p. 238). Instruments should therefore be of the highest quality, which would make it easier to obtain valid and reliable evidence [7]. In this sense, the usual way to assess the quality of an instrument is by consulting experts, which fundamentally consists of evaluating an instrument using a procedure known as expert judgement [8].

Content validation through expert judgement is defined by Escobar-Pérez & Cuervo-Martínez [9] (p. 29) as an informed opinion from individuals with a track record in the field who are regarded by others as qualified experts and who can provide information, evidence, judgements, and assessments. Evaluation through expert judgement consists of asking a number of individuals to make a judgement on an instrument or to express their opinion on a particular aspect [10]. Content validations are generally conducted either during the design of a test or for the validation of the translation and standardization of an instrument for use in a different culture. In both cases, the role of experts is fundamental in clarifying, adding, and/or modifying the necessary aspects [11].

In order to validate the content of an instrument through expert judgement, content validity and expert judgement must be conceptualized, an implementation procedure must be established, and statistical alternatives for data analysis must be provided in order to make decisions [9]. To ensure their proper implementation, various aspects should be taken into account, such as the criteria and strategies for the selection of experts, the appropriate number of experts, and the instruments involved. As mentioned in the previous paragraph, the role of experts is fundamental in this endeavor, as it allows for nuances to be added or modified, which is why the diversity of experts is important. For the selection of the judges, the aforementioned procedure is used. It is important to select individuals who are knowledgeable in the subject matter, either because of their academic background, work experience, or recognition in the community [9]. When selecting the judges, it is important to choose individuals who are knowledgeable about the subject, either because of their academic background or because of their work experience. The information provided by the judges may be collected individually, in groups, or using the Delphi method [10]. As part of the procedure, the experts assess the different dimensions using a numerical scale [12] while taking into consideration their reasoned opinions. This is useful to identify the weaknesses and strengths of the instrument [5]. As a result, the expert judgement includes both quantitative and qualitative aspects.

According to Cabero and Llorente [10], expert judgement as an evaluation strategy offers many advantages, such as the high quality of the judges’ responses and the possibility of obtaining extensive information on the subject matter. This is a procedure whose correct performance is sometimes the only indicator of the content validity of a research instrument [9]. Therefore, using formal methods leads to more scientifically sound results, especially when placing particular emphasis on the characterization and selection of experts, on the use of a scale that facilitates quantitative assessments, and on the analysis of the results using appropriate statistical tests [12].

Usually, the nutritional knowledge, beliefs, and habits of pregnant women are measured because their nutritional needs increase during this period. In addition to fulfilling the mother’s needs, the needs of the fetus must also be covered [13,14]. Inadequate nutritional intake at this stage in life may have negative short- and long-term health consequences for both the mother and the child [2,15,16]. Pregnancy is widely regarded as a vulnerable nutritional stage. As a consequence, it is of the utmost importance to identify any existing problems through validated instruments in order to be able to address them using suitable educational measures. Knowing which supplements are useful during pregnancy and how much weight to gain during this period is very important when it comes to introducing proper eating behaviors to pregnant women. Nutrition-related myths and the deficit of nutrition education put at risk the presence of essential foods in their diet and may lead to increased consumption of foods with low nutritional value and quality [17].

Behaviors are difficult to modify and are generally influenced by various environmental factors beyond personal control [18]. However, it has been shown that individuals can regulate their eating behaviors for different reasons [19]. Pregnancy may be one of these reasons, as it is a motivating period of time to acquire healthy eating patterns. The dietary behaviors of pregnant women have been found to be different compared to those of non-pregnant women [20].

Eating is not only a nutritional phenomenon, but also a cultural and social phenomenon where different beliefs influence the acceptance or rejection of certain foods [21]. Most religions establish rules about the intake of certain foods, which foods are to be considered pure or impure, the times established of fasting, etc. [22]. It should not be forgotten that nutritional interventions targeting pregnant migrants should also consider the symbolic nature of food [23].

Pregnancy is thus a highly vulnerable period in terms of nutrition, as well as a motivating stage in life for modifying eating behaviors, and pregnant women sometimes have poor nutritional knowledge. For these reasons, healthcare and teaching staff need a useful instrument for measuring their level of nutritional knowledge and their nutritional beliefs and habits.

Different studies aim to assess nutritional knowledge and beliefs during pregnancy and provide useful information on nutritional knowledge and beliefs [3,24]. However, these studies focus on conducting such assessments using methods which have already been validated. In contrast, the present study also presents the appropriate tools to validate a questionnaire, which is why this study uses a more innovative method.

Having this new tool available would be very useful for healthcare providers in identifying the gaps in nutritional knowledge and the unhealthy eating habits and misconceptions held by pregnant women from different cultures. This tool will thus make it possible to design nutritional education strategies to promote healthy eating behaviors while taking into account socio-cultural aspects [25,26], eating habits, and levels of education [27].

The objective of this study is to present the content validation process, using expert judgement, of an instrument for measuring two dimensions. The first dimension is nutritional knowledge and the nutritional beliefs and the second is eating habits of pregnant women from different cultures in the city of Melilla, Spain, where the crude birth rate was 15.95 births per 1000 inhabitants in 2018 [28] compared to 7.86 at the national level [29].

## 2. Materials and Methods

This is a descriptive, psychometric study on content validity through expert judgement which was conducted at the Melilla Campus of the University of Granada (Spain).

### 2.1. Sample

Convenience and intentional sampling were used. The participants were 14 doctors (PhD degree holders) from the University of Granada (Spain), with a mean work experience (in research and teaching) of 14.92 years (SD: 10.37 years) and academically trained in the fields of educational psychology, language and literature didactics, research and diagnosis methods in education, nursing, obstetrics and gynecology, and nutrition and food science (Table 1).

### 2.2. Instrument

Many pregnant women have no knowledge of the recommended guidelines for weight gain [30] or when to start taking folic acid [31]. Pregnant women generally have limited knowledge of dietary guidelines for eating healthily during pregnancy [32], which is why it is important for pregnant women to be aware of the issues raised in this study. With regard to the selection of questions in the questionnaire, it should be noted that the questions have been designed with the aim of assessing whether pregnant women are aware of the most relevant aspects of their nutrition and the proper development of their pregnancy. To this end, we have taken into account aspects dealt with in maternal education courses, criteria of interest included in dietary recommendations or food guides for an adequate nutritional status in pregnant women, and further consulted literature [2,14,16,17,33]. The content questions regarding nutritional knowledge, beliefs, and habits were selected after conducting a literature review using the PubMed and Web of Science databases, as well as guidelines within the framework of food institutions. After this search, 20 questions were proposed and scored according to four categories.

The questionnaire “Nutritional knowledge, beliefs, and habits during pregnancy” (NKBHP) consists of two parts or dimensions (nutritional knowledge and nutritional beliefs/habits) with 10 items each. Each item was assessed following the “Template for assessing content validity through expert judgement” developed by Escobar-Pérez and Cuervo-Martínez [9], which establishes four levels (“does not meet the criterion,” “low level,” “moderate level,” and “high level”) for each one of the characteristics assessed. These characteristics are sufficiency, clarity, coherence, and relevance (Table 2). The indicator “one” was assigned when the item did not conform to the category, up to indicator “four”, which was assigned when the item fully conformed to the category (only sufficiency was scored by dimension rather than by item). The experts’ qualitative observations for each of the twenty items that made up the initial instrument were also taken into account.

### 2.3. Statistical Analysis

The SPSS Statistics 24.0 software was used for data analysis. The degree of agreement among the experts was determined using Fleiss’ *κ*, as this is an analytical statistic that makes it possible to assess the degree of agreement among three or more raters who independently judge a series of items using an instrument with a certain number of ordinal categories [34,35]. The minimum value assumed by this coefficient is 0 and the maximum value is 1. The scale produced by Landis and Koch [36], which quantitatively expresses the strength of agreement among observers, was used for the interpretation of Fleiss’ *κ* values (Table 3).

### 2.4. Procedure

The sample was selected using convenience (or affinity) sampling. The experts participated voluntarily and signed the informed consent form. All of them were experts in areas that could contribute to improving both the content, procedural, and wording aspects of the questionnaire. A cover letter was sent to the judges by email with acknowledgement of receipt alongside the questionnaire to be validated. This letter contained information on the main objective of this study and how to respond and assured the experts of the confidentiality of their data. In order for the experts to evaluate a certain number of items, both the amount of information and the way in which it is presented are important [37]. These aspects were therefore all taken into account. The researchers were available at all times to answer any questions the experts might have had. The judges sent their signed informed consent forms by mail and were given one month to assess and rate the questionnaire online. All the experts who were sent a request agreed to participate. No reminders had to be sent to them, as they all responded within the deadline.

Once the experts had assessed the questionnaire, the resulting instrument was subjected to the standard pre-test using potential respondents in order to have information on how it would work in real life [10]. To this end, a dichotomous yes/no response was encoded in each of the items to measure their degree of understandability and applicability/feasibility. The sample consisted of 102 women of childbearing age from various cultures and religions (50% Muslim, 46% Christian, 3% Jewish, and 1% Hindu) from the city of Melilla (Spain). This distribution was based on a demographic study by the Union of Islamic Communities of Spain [38]. The questionnaires were administered at the healthcare centers and were completed on-site in person, and all the questionnaires were returned. The sampling was incidental, with the participation of women who were visiting the healthcare center for various health-related issues. The time taken by the participants to answer the questionnaire ranged from 5 to 10 min. This was assessed quantitatively. We simply collected the questionnaires and subsequently analyzed the responses. Degrees of understandability were classified as follows: high understandability (equal to or greater than 85%), medium understandability (from 80% to 85%), and low understandability (less than 80%).

### 2.5. Ethics

This research was conducted in compliance with the ethical principles set out in the Declaration of Helsinki. All participants were informed of the purpose of this study and participated voluntarily, having signed an informed consent form. The knowledge and approval by management of the Comarcal Hospital of Melilla, on which the Unit for Attention to Women depends, was assured.

## 3. Results

### 3.1. Content Validation by Expert Judgement

For the evaluation of the original instrument, the proportion of possible agreements occurring in each dimension was taken into account in the calculation of Fleiss’ *κ*. The magnitude of the strength of agreement was considered to be *almost perfect* for both dimensions by the set of judges, as shown in Table 4.

The magnitude of the strength of agreement by pairs of experts was also analyzed. Values corresponding to “substantial” and “almost perfect” were found, as shown in Table 5.

In addition, the characteristics of the instrument regarding the indicators of sufficiency, clarity, coherence, and relevance were assessed using the ordinal measurement scale. A strength of agreement between “substantial” and “almost perfect” was found (Table 6), with “relevance” having the highest values in both dimensions (0.890 in knowledge and 0.901 in habits) based on the degree of overall agreement among the judges.

The statistical significance threshold for the results was set at *p* < 0.05, with a 95% confidence interval for all cases. Agreement on the characteristic of “relevance” was found to be statistically significant (*p* < 0.001) for both dimensions. Agreement on “sufficiency” was also found to be statistically significant (*p* < 0.001) for the dimension of nutritional beliefs and habits.

These results, together with the qualitative observations and recommendations made by the judges on the items included in the two dimensions, made it possible to keep the original number of items at 20. However, the wording of five of the items was amended, resulting in the final validated instrument.

### 3.2. Measurement of Applicability: Pre-Test

The final validated instrument was administered to a total of 102 women of childbearing age to determine the percentage of comprehensibility of the dimensions and their corresponding items. The degree of comprehensibility of the instrument was found to be in the highest range, at 99.7%, as shown in Table 7.

## 4. Discussion

Nutrition and health professionals and researchers need valid and reliable behavioral measures that are appropriate for use in a variety of community settings [39]. The validation of an instrument is an on-going and dynamic process that becomes more consistent the more psychometric properties are determined for that particular instrument in different contexts and populations. Validation will also be determined by the type and purpose of the instrument. In this case, where the aim is to collect factual information related to the knowledge and practices of certain subjects, content validity by experts takes priority [4,40].

The content validity of an instrument refers to the degree to which this instrument covers an adequate sample of the contents it is intended to cover, without omissions, oversights, or imbalances [41]. However, an instrument does not have to cover in detail each of the areas that make up a concept, as this would result in an overly large instrument. The instrument must therefore contain a representative sample of domains and possible issues relating to the concept of interest [42]. The twenty items of the questionnaire presented here include the most relevant aspects for determining the nutritional knowledge, beliefs, and habits of pregnant women from different cultures.

Even though ensuring the content validity of an instrument may seem to be time consuming and costly in terms of human resources, it deserves greater attention when developing a valid assessment instrument [43]. The evaluation technique of expert judgement can be very useful for the validation of diagnostic instruments but requires the correct selection of experts [10].

Determining the number of experts that should be involved in the content validation is one of the main difficulties to be addressed, as there is no widespread consensus on this [11]. Availability and level of knowledge on the subject matter of the research are some of the criteria used to establish the sufficient number of experts [10]. However, the appropriate number of experts will depend on the method used. Some methods are designed to measure agreement between two judges [37]. Other methods require a higher number of experts, between 7 and 30 [44,45]. Rubio et al. [46] propose a range of 6 to 20 experts and establish that using a greater number of experts may generate more information on the measure in question. In general, many authors recommend more than 10 experts [12,47,48,49]. As a result, a total of 14 experts were selected for this study.

With respect to experience, it is recommended that at least two of the judges be measurement and evaluation experts [9]. The current study includes two experts in the field of research and diagnosis methods in education.

The selection of experts is another important consideration. There are various procedures for this, such as structured procedures including selection criteria (e.g., graphical biographies and competence coefficients), and unstructured procedures without selection filters, e.g., the closeness or affinity of the researchers to the judges [10]. The latter procedure, the closeness (or affinity) of the researchers to the experts, has been used in this research.

In this study, experts were selected on published criteria while considering a procedure that ensures the assertiveness of their assessments. The criteria were the following: the judges’ experience in issuing judgements and decision-making; their academic and scientific reputation; their willingness and motivation to collaborate; their objectivity; their compliance with what has been established [9]; and their ability to perform the question classification techniques required to validate the content [50]. Following this procedure, experts complying with these characteristics were sought to prevent introducing content bias in the analysis of the data.

The quality of the results in a study using expert judgement is strongly related to the experts selected. Therefore, using a good selection procedure is of paramount importance [51]. Ténière-Buchot [52] reports that there are three types of experts: tactical experts, conciliatory experts, and communicative experts. Tactical experts are selected on the basis of their experience and knowledge of the subject matter. Conciliatory experts are selected for their objectivity and common sense. Communicative experts are the experts who are most involved in the study. In this case, tactical experts or specialists were included, since, according to Ténière-Buchot [52], specialists in the field ensure a higher scientific quality of the study.

Another aspect to consider in content validation through expert judgement is the amount of time given to judges to make their judgement [5]. In this study, a one-month deadline was established for each of the judges to analyze the weaknesses and strengths of the instrument and submit their opinion online.

Content validation requires the participation of both researchers and members of the target population [40]. The current study involved both experts who are linked to the research field and to the methodological aspects of the instrument, as well as potential members of the target population, since it was women of childbearing age who underwent the pre-test procedure. Recently published studies use this procedure for the content validation of instruments. In these studies, in addition to the expert phase, the instrument designed is subsequently subjected to a pre-test where a focus group assesses each item for clarity taking into account the level of understandability of the instrument [53]. Similarly, in a study by Bernal-García et al. [54] a pre-test was subsequently conducted to measure the degree of understandability of the instrument, which turned out to be high-ranking, as in this study.

Regarding the statistical analyses to calculate the agreement between the judges, the *κ* statistic and Kendall’s coefficient are the most widely used [9]. In this study, the *κ* statistic was used, as it provides quantifiable methods to assess judgments on content and has the additional possibility of eliminating random chance agreement [55]. The *κ* statistic can be used to assess the degree of agreement at the individual level [39], although this is not how it is used in this study.

Given that there were multiple raters in this study, the Fleiss’ *κ* statistic was used, as it is based on the agreement between different pairs of raters, which increases the accuracy of the results [35,56,57,58], unlike the weighted Cohen’s *κ* statistic, which is used for nominal variables in the case of two raters [59].

For the questionnaire analyzed, the Fleiss’ *κ* statistic yielded an “almost perfect” strength of agreement for each dimension and a strength of agreement between “substantial” and “almost perfect” for pairs of experts. Similar results were obtained by Bernal-García et al. [54]. However, in this study, the strength of agreement by pairs of experts attained a somewhat lower level, between “moderate” and “almost perfect”.

The rating of the relevance, clarity, simplicity, and ambiguity of items using four-point scales is something that has been going on for years for content validation [33,43,60,61]. The same parameters were considered for this study. However, instead of simplicity, sufficiency was considered, as indicated in a study by Escobar-Pérez and Cuervo-Martínez [9].

In healthcare research, many relevant results and variables of interest are abstract concepts known as theoretical constructs. The use of valid and reliable instruments to measure such constructs is an essential component of the quality of research [62].

Several studies enquire whether pregnant women have received information on healthy eating habits during pregnancy [3,24,63,64]. This question has been included in the present questionnaire to obtain this information, which is considered to be important when assessing nutritional knowledge.

Other studies determine dietary knowledge and beliefs during pregnancy using food consumption surveys [65,66,67]. In the case of the present study, the aim is to validate a questionnaire based on nutritional knowledge and beliefs and not just based on food consumption with a quantitative approach. 

There are numerous methods for measuring food-related aspects. However, almost all of them focus on studying food consumption from a quantitative approach. If this quantitative information is combined with the assessment of eating behaviors, such as nutritional beliefs and habits, the result would be a more complete study of the eating process in the different subjects, which could facilitate making nutritional educational recommendations [68].

Rather than in designing new tools, there is now interest in establishing indices that provide information on specific behavioral patterns associated with eating habits and socio-cultural habits, as well as on nutrients and foods consumed [69]. Culture is therefore an important factor to take into account when it comes to understanding eating behaviors [70,71].

As for the limitations of this study, it is worth noting that the participants in the pre-test were women of childbearing age and not pregnant women. As a consequence, this test cannot be interpreted in its real context, and as these were quantitative questions, no in-depth qualitative data could be obtained from these participants.

With respect to content validity through expert judgement, there are aspects that the researchers cannot control for, such as the complexity or degree of difficulty of the task. It should be noted that even when a test receives a very good rating from the experts, it must be continually reviewed and improved [9]. Furthermore, this process requires considerable attention and formal methods for the selection of experts, for the use of a scale that facilitates quantitative assessments, and for the analysis of the results using relevant coefficients. In general, validation processes through expert judgement prove to be demanding and time consuming.

## 5. Conclusions

Although content validity is subjective, it can add objectivity to the study by using statistics such as Fleiss’ *κ*, which is very useful for measuring agreement between experts and thus to be able to validate the instrument correctly. Complementing this with the pre-test procedure described also facilitates the determination of the degree of comprehensibility of the final instrument. Understanding the need for and the process of conducting content validation studies is important for healthcare professionals and researchers. Having a guide in place may prove very helpful. We believe the objective of validating a measurement instrument has been met. This instrument may be used in different populations of pregnant women to determine their nutritional knowledge, beliefs, and habits using new psychometric tests. These tests will increase the validity of the instrument and favor comparisons between different populations in Spain and in other Spanish-speaking countries. This in turn will establish educational measures to ensure adequate eating behaviors among pregnant women and thus prevent negative health consequences for both the mother and the future baby. It is therefore considered of great utility to have reliable tools available that may be targeted at different cultures, since culture influence eating behaviors of the population in general and of pregnant women in particular.

## Figures and Tables

**Table 1:** Judges, field of expertise, academic training, and work experience.

Judge	Field of Expertise/Academic Training	Work Experience (Years)
1	Educational psychology	22
2	Educational psychology	5
3	Language and literature didactics	11
4	Language and literature didactics	19
5	Research and diagnosis methods in education	9
6	Research and diagnosis methods in education	18
7	Nursing	32
8	Nursing	10
9	Nursing	14
10	Nursing	6
11	Obstetrics and gynecology	3
12	Obstetrics and gynecology	23
13	Nutrition and food science	35
14	Nutrition and food science	2

**Table 2:** Categories and indicators used by the judges to validate the tool.

Categories	Indicators
**Sufficiency** The items within the same dimension suffice to measure this dimension	The items are sufficient to measure the dimension
	The items measure some aspects of the dimension, but do not represent the full dimension
	A few items must be added in order to fully assess the dimension
	The items are insufficient
**Clarity** The item can be understood easily, i.e., syntax and semantics are appropriate	The item is unclear
	The wording of the item requires several modifications or a very large modification in terms of meaning or word order
	Some of the terms in the item require very precise modificationsThe item is clear, with appropriate semantics and syntax
**Coherence** The item is logically related to the dimension or indicator it is measuring	The item bears no logical relationship to the dimension
	The item has a tangential relationship to the dimension
	The item has a moderate relationship to the dimension it is measuring
	The item is completely related to the dimension it is measuring
**Relevance** The item is essential or important, i.e., it must be included	The removal of the item would not affect the measurement of the dimension
	The item is somewhat relevant, but another item may be covering what this item is measuring
	The item is rather important
	The item is very relevant and should be included

Source: adapted from Escobar-Pérez and Cuervo-Martínez [9] (p. 37).

**Table 3:** Fleiss’ *κ* values and strength of agreement [36].

Fleiss’ *κ*	Strength of Agreement
0.00	Poor
0.1–0.20	Slight
0.21–0.40	Fair
0.41–0.60	Moderate
0.61–0.80	Substantial
0.81–1.00	Almost perfect

**Table 4:** Strength of agreement among judges for the dimensions of the original instrument.

Dimensions	Fleiss’ *κ*	Strength of Agreement (Landis and Koch, 1977)
Knowledge	0.860	Almost perfect
Beliefs and habits	0.830	Almost perfect

**Table 5:** Agreement by pairs of experts.

Dimensions	Fleiss’ *κ*—Agreement by Pairs of Experts													
	1–14	2–13	3–12	4–11	5–10	6–9	7–8	8–6	9–5	10–4	11–3	12–2	13–1	14–7
Knowledge	0.929	0.868	0.763	0.885	0.717	0.920	1	0.830	0.811	0.785	0.900	0.735	0.889	1
Beliefs and habits	1	0.984	0.711	0.700	0.732	0.833	0.931	0.846	0.744	0.714	0.706	0.708	0.949	1

**Table 6:** Fleiss’ *κ* and statistical significance of the characteristics of the original instrument.

Dimensions	Characteristics	Fleiss’ *κ*	*p*
Knowledge	Sufficiency	1	0.001
	Clarity	0.805	0.002
	Coherence	0.795	0.030
	Relevance	0.890	<0.001
Beliefs and habits	Sufficiency	1	<0.001
	Clarity	0.780	0.037
	Coherence	0.847	0.007
	Relevance	0.901	<0.001

**Table 7:** Percentages of comprehensibility of the dimensions and their items in the final version of the validated instrument.

Dimensions	Items	Degree of Comprehensibility (%)	
		Yes	No
Knowledge	Maximum weight gain	98	2
	Initiation of folic acid consumption	100	0
	What folic acid is for	100	0
	Iron	99	1
	What iron is for	100	0
	Instructions from healthcare personnel	100	0
	What to do during pregnancy	100	0
	Fiber during pregnancy	100	0
	Salt during pregnancy	99	1
	Fluids	98	2
Beliefs and habits	Awareness	100	0
	Number of meals and times	100	0
	Influence	100	0
	Time spent eating	100	0
	Most hungry	100	0
	Diet	100	0
	Good eating habits	100	0
	Importance of food	100	0
	Harmful foods	100	0
	Beneficial foods	100	0
